# 肺结节评估四大指南比较分析

**DOI:** 10.3779/j.issn.1009-3419.2017.07.08

**Published:** 2017-07-20

**Authors:** 春全 刘, 永 崔

**Affiliations:** 100050 北京，首都医科大学附属北京友谊医院胸外科 Department of Thoracic Surgery, Beijing Friendship Hospital, Capital Medical University, Beijing 100050, China

**Keywords:** 肺结节, 共识, 指南, Lung nodules, Consensus, Guideline

## Abstract

近20年来，随着计算机断层扫描（computed tomography, CT）技术的提高和肺癌高危人群筛查的普及，越来越多的肺部小结节被发现，然而肺结节的定性诊断仍有很多困难。肺结节是临床上一种常见的现象，恶性结节早期发病比较隐匿，如果不进行早期干预，其病程迅速、恶性程度强、预后差。如果能在早期阶段对病灶进行手术切除，将会明显改善肺癌患者的预后。目前针对肺结节的处理指南层出不穷，但各大指南均未达成统一的共识。本文拟对在国内影响最大的四个指南：美国国家综合癌症网络非小细胞肺癌（non-small cell lung cancer, NSCLC）临床实践指南、美国胸科医师协会肺癌诊疗指南、Fleischner-Society肺结节处理策略指南、肺结节的评估亚洲共识指南所推荐的肺结节诊断和处理策略进行介绍和分析。

## Abstract (en)

In the past 20 years, with the popularization of low-dose computed tomography (CT) screening, detection rate of lung nodules increased significantly. However, there are still many difficulties in making qualitative diagnosis for pulmonary nodules. The Lung nodule is a clinical common lung disease. The early onset for malignant nodules is quite hidden. Without early intervention, the course of disease can develop rapidly. For malignant nodules, the exacerbation can be very severe. Besides, the therapeutic effect can be unsatisfactory. If the lesion resection can be performed in early stage, lung cancer patients' prognosis can be improved significantly. At present, the guidelines of lung nodules' treatment are diverse, but these guidelines still can't reach a consensus until now. This article reviews the literature in National Comprehensive Cancer Network (NCCN) Clinical Practice Guidelines in Oncology for Non-Small Cell Lung Cancer (NSCLC), American College of Chest Physicians (ACCP) guidelines for the diagnosis and treatment of pulmonary nodules, Fleischner society lung cancer treatment strategy guide, and clinical practice consensus guidelines for Asia, which are pertaining to lung nodules diagnosis and treatment strategy and try to explain the similarities and differences between them.

肺结节是一种临床中常见的现象，包括良性结节和恶性结节，恶性肺结节早期发现比较隐匿，如果不早期干预，其病程迅速、恶性度强、预后差。目前对肺结节的定性诊断仍有很多困难，在临床外科切除的肺结节中，30%左右为良性的，所以正确评价肺结节的良恶性，有助选择正确的治疗手段^[1]^。随着影像学技术的发展以及低剂量计算机断层扫描（low-dose computed tomography, LDCT）的普及，肺结节的检出率明显增高，肺结节的临床处理与决策逐渐成为困扰临床医生的问题之一。2013年美国癌症协会发布的最新报告显示，美国肺癌5年整体存活率为16%，然而，由于早期筛查在美国的开展，早期肺癌5年生存率已达70%-90%^[2, 3]^。因此，国内外的专家们一直想通过对肺癌的筛查来实现早期诊断及治疗，从而降低死亡率^[4-9]^。目前针对肺结节的处理指南层出不穷，但各大指南均未达成统一的共识。本文对美国国家综合癌症网络（National Comprehensive Cancer Network, NCCN）、Fleischner学会、美国胸科医师协会（American College of Chest Physicians, ACCP）和亚洲共识四大指南进行分析，进一步阐释肺结节诊断和处理策略的异同并对其进行综述。

## 1 四大指南肺结节影像学随访策略的区别

影响肺结节的随访策略的主要因素是肺结节的影像学特点以及患者自身的危险因素，影像学因素包括肺结节的大小、形状、密度、数量、肺实质异常以及正电子发射型计算机断层显像（positron emission computed tomography, PET-CT）中的氟脱氧葡萄糖（fluorodeoxy-glucose, FDG）活性，其中最主要的是与之前影像学资料进行比较，评估肺结节的稳定性，影响患者自身的危险因素主要是吸烟史和年龄。四大指南对于肺结节评估筛查的目标人群非常相似，但对于阳性结果的CT随访策略却又有不同之处，且对于筛查风险问题仍存在争议。

### 1.1 对于实性结节影像学随访策略的区别（表 1）

**1:** 实性结节影像学随访策略的区别 The difference of the follow up strategy of solid nodules

Item	Low risk (mm)					High risk (mm)			
	＜4	＞4 to＜6	＞6 to＜8	≥8		＜4	＞4 to＜6	＞6 to＜8	≥8
2016 NCCN Guidelines	No follow-up needed	CT at 12 mo, if stable, no further follow-up	CT at 6-12 mo, if stable, then repeat CT at 18-24 mo	CT at 3, 9, and 24 mo, consider PET or biopsy		CT at 12 mo, if stable, no further follow-up	CT at 6-12 mo, if stable, repeat CT at 18-24 mo	CT at 3-6 mo, if stable, repeat CT at 9-12 mo and 24 mo	CT at 3, 9, and 24 mo, consider PET or biopsy
2017 Fleischner Society Guidelines	No routine follow-up	No routine follow-up	CT at 6-12 mo, if stable, then repeat CT at 18-24 mo	Consider CT, PET, or tissue sampling at 3 mo		Optional CT at 12 mo	Optional CT at 12 mo	CT at 6-12 mo then CT at 18-24 mo	Consider CT, PET or tissue sampling at 3 mo
2013 ACCP Guidelines	No follow-up needed	CT at 12 mo	CT at 6-12 mo, if stable, then repeat CT at 18-24 mo	CT at 3, 9, and 24 mo, consider PET or biopsy		No follow-up needed	CT at 6-12 mo, if stable, repeat CT at 18-24 mo	CT at 3-6 mo, if stable, repeat CT at 9-12 mo and 24 mo	CT at 3, 9, and 24 mo, consider PET or biopsy
2016 Clinical practice consensus guidelines for Asia	Annual CT surveillance	CT at 12 mo and then annual CT surveillance	CT at 6-12 mo, if stable, then repeat CT at 18-24 mo and then annual CT surveillance	CT at 3-6, 9-12, and 18-24 mo, if the nodule sclear growth then surgical biopsy		Patient discussion	CT at 6-12 mo, if stable, then repeat CT at 18-24 mo and then annual CT surveillance	CT at 3, 6, and 12 mo then annual CT surveillance	PET scan if hypermetabolic，surgical biopsy, if surgical biopsy is positive, then surgical resection
CT: computed tomography; NCCN: National Comprehensive Cancer Network; ACCP: American College of Chest Physicians; PET: positron emission computed tomography.									

#### 1.1.1 ＜4 mm

NCCN、ACCP、Fleischner协会指南均建议不需要随诊，亚洲共识指南建议每年复查1次CT。

#### 1.1.2 ＞4 mm且＜6 mm

NCCN协会指南建议低危人群1年后复查CT；建议高危人群半年到1年之间、1年半到2年之间复查2次CT^[10]^。ACCP指南处理方案与NCCN大致相同，只是人群分类标准不同，ACCP指南中把人群分为有、无肺癌危险因素，而NCCN和Fleischner协会则根据危险因素的多少，更加详细的分为低风险人群和高风险人群。Fleischner协会指南建议不需常规随访。亚洲共识指南建议低危人群每年复查CT，中、高危人群在NCCN指南基础上每年复查1次CT。

#### 1.1.3 ＞6 mm且＜8 mm

NCCN指南建议低危人群在半年到1年之间、1年半到2年之间复查2次CT，建议高危人群3个月到半年之间、9个月到1年之间、2年复查3次CT。ACCP指南处理方案与NCCN大致相同。Fleischner协会指南建议半年到1年复查CT，之后再考虑1年半到2年之间复查CT。亚洲共识指南建议低危人群半年到1年之间、1年半到2年之间复查2次CT，以后每年复查1次CT；中、高危人群分别在第3个月、半年、1年复查3次CT，以后每年复查1次CT。

#### 1.1.4 ≥8 mm

NCCN和ACCP指南均建议在第3个月、第9个月、2年复查3次CT，可行动态增强CT、PET和（或）活检^[11]^。Fleischner协会指南建议3个月后复查CT、PET-CT或活检。亚洲共识指南建议低危人群3个月到半年之间、9个月到1年之间、1年半到2年之间复查3次CT，若肺结节较前增大，则建议外科活检；建议中危人群行PET-CT扫描，高度怀疑者可行外科活检，中低度怀疑者建议定期监测；建议高危人群行外科手术活检，若活检结果为阳性，建议手术切除。

### 1.2 对于磨玻璃和部分实性结节影像学随访策略的区别（表 2）

**2:** 磨玻璃和部分实性结节影像学随访策略的区别 The difference of the follow-up strategies between the ground-glass nodules and part solid nodules

Item	Ground-glass nodules (mm)				Part-solid nodules (mm)			
	＜5	≥5 to＜6	≥6		＜5	≥5 to＜6	≥6 to＜8	≥8
2016 NCCN Guidelines	No follow-up needed	CT at 3 mo, and annual CT for at least 3 years	CT at 3 mo, and annual CT for at least 3 years		CT at 3 mo, and annual CT for at least 3 years	Biopsy or surgical resection	Biopsy or surgical resection	Biopsy or surgical resection
2017 Fleischner Society Guidelines	No follow-up needed	No follow-up needed	CT at 6-12 mo to confirm persistence, then CT every 2 years until 5 years		No follow-up needed	No follow-up needed	CT at 3-6 mo to confirm persistence, if unchanged and solid component remains 6 mm, annual CT should be performed for 5 years	CT at 3-6 mo to confirm persistence, if unchanged and solid component remains 6 mm, annual CT should be performed for 5 years
2013 ACCP Guidelines	No follow-up needed	Annual CT for at least 3 years	Annual CT for at least 3 years		CT at 3, 12, 24 mo, and then annual CT for at 1-3 years	CT at 3, 12, 24 mo, and then annual CT for at 1-3 years	CT at 3, 12, 24 mo, and then annual CT for at 1-3 years	CT at 3 mo to confirm, persistencIf persistent, biopsy surgical resection if a nodule ＞15 mm at first CT scan，then biopsy, PET or surgical resection
2016 Clinical practice consensus guidelines for Asia	Discuss role of continued surveillance with patient	Annual CT surveillance for at least 3 years, consider ongoing annual CT surveillance after discussion with patient	Annual CT surveillance for at least 3 years, consider ongoing annual CT surveillance after discussion with patient		CT at 3, 12, 24 mo, and then annual CT surveillance	CT at 3, 12, 24 mo, and then annual CT surveillance	CT at 3, 12, 24 mo, and then annual CT surveillance	CT at 3 mo, and consider antimicrobial therapy (nonsurgical or surgical biopsy consider PET scanning for staging before biopsy)

#### 1.2.1 ＜5 mm孤立性纯磨玻璃结节

NCCN、Fleischner协会和ACCP指南均建议不需要随诊^[12, 13]^，亚洲共识指南建议每年复查1次CT。

#### 1.2.2 ≥5 mm孤立性纯磨玻璃结节

NCCN指南建议3个月、1年、2年、3年共复查4次CT。ACCP指南与NCCN指南大致相同，但强调3个月内不需要复查CT。亚洲共识指南建议每年复查1次CT，亚洲共识指南与Fleischner协会指南的区别在于亚洲共识要求连续3年复查CT后，如果结节无明显变化，仍需继续年度复查CT，而Fleischner协会则建议3个月-6个月复查CT，确定病灶是否还存在，如果病灶不变或者实性成分维持在＜6 mm，需每年复查CT，满5年。

#### 1.2.3 孤立部分实性结节

NCCN指南建议结节稳定或实性成分＜5 mm时，3个月、1年、2年、3年复查4次CT；结节稳定或实性成分≥5 mm时，活检或手术切除^[14]^。ACCP指南建议结节≤8 mm时，3个月、1年、2年复查3次CT，然后进行1年-3年的年度随访。建议结节 ＞8 mm时，3个月复查1次CT，如果结节持续存在，需考虑PET、非手术活检或手术切除。如果结节发现时即 ＞15 mm，直接进行PET，非手术活检或手术切除^[15]^。Fleischner协会指南建议结节 ＞6 mm时，3个月-6个月复查CT，确定病灶是否还存在，如果病灶不变或者实性成分维持在＜6 mm，需每年复查CT，满5年；结节≤6 mm时，无需常规随访。亚洲共识指南建议结节≤8 mm时，3个月、1年、2年复查3次CT，然后每年复查1次CT；建议结节 ＞8 mm时，3个月复查1次CT，可行抗炎治疗、手术或非手术方式活检，活检前可先行PET-CT检测。

### 1.3 对于多发亚实性结节影像学随访策略的区别（表 3）

**3:** 多发亚实性结节影像学随访策略的区别 The difference of the imaging follow-up strategy of multiple solid nodules

Item	Multiple subsolid nodules (mm)		
	＜5	≥5 to＜6	≥6
2016 NCCN Guidelines	CT at 2 and 4 years	CT at 3 mo annual CT for at least 3 years	CT at 3 mo annual CT for at least 3 years
2017 Fleischner Society Guidelines	CT at 3-6 mo, if stable, consider CT at 2 and 4 years	CT at 3-6 mo, if stable, consider CT at 2 and 4 years	CT at 3-6 mo, subsequent management based on the most suspicious nodules
2013 ACCP Guidelines	There are no clear guidelines multiple subsolid nodules		
2016 Clinical practice consensus guidelines for Asia	Individual assessments of each nodule consider distant metastasis		

NCCN指南建议纯磨玻璃密度结节≤5 mm时，第2年、第4年复查2次CT；纯磨玻璃密度结节 ＞5 mm且无主病灶时，3个月复查1次CT，如果结节稳定，然后进行1年-3年的年度随访；主要结节为部分实性或实性时，3个月复查1次CT，如果结节稳定，则推荐活检或手术切除^[16]^。Fleischner协会指南建议纯磨玻璃密度结节≤6 mm时，3个月-6个月复查CT，如果稳定，考虑第2年和第4年复查CT；纯磨玻璃密度结节 ＞6 mm时，3个月-6个月复查CT，随后针对最有可疑的结节执行随访原则。ACCP指南目前还没有针对多发性肺结节管理的明确指南。亚洲共识指南建议对每个结节进行单独评估，不排除根治性治疗，酌情考虑病理学确认是否为转移。

## 2 四大指南背景、作者、筛查人群、循证医学证据的比较分析

### 2.1 NCCN指南

NCCN是一个由27个世界领先的癌症中心组成的非盈利联盟，成立于1995年1月31日，致力于提高患者的生活质量、提高疾病治疗的效果。NCCN最初由13个原始成员机构组成，他们基本都是来自临床专业的学科带头人和专家，目的是为卫生服务体系提供有价值的信息资源，现任董事会主席是巴恩斯医院癌症中心和华盛顿大学医学院的Timothy J. Eberlein教授。2016年11月，NCCN发布了2017年第3版非小细胞肺癌指南。通过搜索PubMed数据库获得2015年7月1日-2016年7月1日之间非小细胞肺癌的关键文献，搜索的范围被缩小到人类身上研究的英文文章。文章类型主要包括：各阶段临床试验、准则、荟萃分析、随机对照试验、系统评价和验证研究。指南主要参考全国肺癌筛查试验（National Lung Cancer Screening Trial, NLST），该实验涉及53, 000多名目前或既往重度吸烟者，评估了低剂量CT扫描和胸片对肺癌患者进行检测的的风险和益处，并指出使用低LDCT扫描相对于胸片可以降低肺癌死亡率的20%^[17, 18]^。NCCN、ACCP、美国预防服务工作组（United States Preventive Services Task Force, USPSTF）、欧洲肿瘤医学学会（European Society for Medical Oncology, ESMO）、美国外科医生学会（American College of Surgeons, ACS）和其他组织推荐使用LDCT对高风险人群进行肺癌筛查，并强调LDCT的筛查不能代替戒烟^[19-22]^。NCCN指南提出对肺结节进行评估时，需要胸外科、放射科、呼吸科等多学科共同合作，给出最优的诊断以及后续的治疗措施。病人自身的主要危险因素是吸烟，其与大多数肺癌患者死亡相关^[23-27]^。患者自身已知的危险因素包括年龄、既往的癌症病史、肺癌家族史、接触石棉、氡、铀等^[28-32]^、其他肺部疾病（慢性阻塞性肺病、肺纤维化）^[33, 34]^、传染性病原体接触（如真菌感染、结核病疫区）、表明感染的高危因素病史（如免疫抑制、呼吸、传染性呼吸道症状）等。风险评估虽然可以用来衡量个人危险因素和影像学的差异，但不会取代多学科评估对肺癌的诊断。

### 2.2 Fleischner Society指南

Fleischner协会是一个国际多学科胸部放射学医学会，主要研究胸部疾病的诊断和治疗。协会于1969年由8名胸部影像学医生创建，主要成员包括成人和儿科放射学专家，病理学家、胸外科医生、生理学家、形态学家、流行病学及其他相关科学专家。协会目前大约有70名成员，其中40名为高级成员，主席是William D. Travis教授。2005年Fleischner学会发表了肺实性结节的处理指南，目前已经广泛用于临床^[35]^。然而，越来越多的人意识到其非实性肺结节处理方案的不足。Fleischner学会总结了非实性肺结节的定义，并且查找2011年初由国际肺癌研究协会（International Association for the study of lung cancer, IASLC）、美国胸科学会（The American Thoracic Society, ATS）、欧洲呼吸学会（The European Respiratory Society, ERS）联合公布的肺腺癌的国际多学科分类标准，结合相关文献进行分析^[36-42]^，2013年Fleischner学会发布了6条推荐指南，其中3条是孤立性非实性肺结节的处理方案，另外3条是针对多发亚实性肺结节。该指南对许多非实性肺结节研究的文献进行系统性回顾总结。2013年与2005年关于肺结节的处理指南相比有以下区别：新版指南没有区分吸烟者、未吸烟者或戒烟者，没有单独区分肺癌家族史和暴露于潜在的致癌因子，并且将多发非实性肺结节纳入其中。2017年Fleischner学会重新修订了肺结节评估指南，本次修订是根据NLST、荷兰-比利时肺癌筛查研究（Dutch-Belgian Randomized Lung Cancer Screening Trial, NELSON）、国际早期肺癌行动计划（International Early Lung Cancer Action Program, iELCAP）、加拿大肺癌早期检测研究以及大不列颠哥伦比亚癌症办事处的肿瘤筛查研究，所有研究数据支持对肺结节采取更温和的处理，本次修订同时涵盖了2005年和2013年的内容，并在其基础上进行了改动，并指出临床中对指南的应用需要更灵活的临床管理路径。

### 2.3 ACCP指南

ACCP成立于1935年，由世界各地的内科医师、医学博士、从事健康工作的人员组成的非营利性的组织，拥有全世界100多个国家的19, 000个成员代表。学会成立的同年创建了医学期刊《*Chest*》，现为月刊，被世界胸科医学界公认为发行量最大、最有影响力的医学杂志之一，致力于肺病学、胸部外科、心脏病学、气道疾患、移植、呼吸与睡眠等方面，2015年-2016年影响因子：5.940。胸部慈善基金会于1996年成立，早期主要关注年轻女性的烟草预防，并在过去的20年里产生重大影响。2003年第一版ACCP指南分析了使用胸片（chest X-ray, CXR）和痰分析进行肺癌筛查实验的相关数据^[43, 44]^，因为胸片、痰液分析对肺癌的筛查已经被大型随机试验和三个系统性回顾研究所证实有效^[45-48]^，血液检测、荧光支气管镜、呼出气体分析等没有作为筛查方式，因为这些指标还有待进一步证实。2007年第二版ACCP指南认为当时尚无明确的循证医学表明存在一种肺癌筛查方式能够降低死亡率，所以不推荐使用LDCT用于肺癌的早期筛查。2013年第三版ACCP指南通过搜索和提取分析ACCP与NCCN、ACS、美国临床肿瘤学会（American Society of Clinical Oncology, ASCO）合作的数据，进行对胸片、痰液分析、低剂量CT的患者死亡率进行比较，最终确定以LDCT作为筛查方式^[49]^。指南回顾从1996年-2011年运用LDCT进行肺癌筛查系统回顾数据^[41, 50-56]^，数据包括8项随机对照试验和13项前瞻性队列研究^[57, 58]^，其中只有3项随机对照试验能够用来评估LDCT筛查率的对肺癌患者死亡率的影响。NLST结果表明，使用LDCT筛查肺癌相比于CXR可以使死亡率减少20%。丹麦的肺癌筛查试验（Danish Lung Cancer Screening Trial, DLCST）和意大利的肺癌筛查试验（Detection and Screening of Early Lung Cancer, DANTE）表明使用LDCT筛查对肺癌患者的生存率并没有影响。三个实验结果不同的原因可能是后两个筛查实验数据少、随访时间短，而且并没有专门对具有高危因素患者进行筛查。指南主要参考NLST的筛查结果，并推荐对高危人群进行LDCT筛查，高危人群的界定标准为55岁-74岁之间，每年吸烟至少30包，目前正在吸烟或戒烟不超过15年。指南最后强调运用LDCT进行肺癌筛查应该在一个有条理、全面、多学科合作的特定的队列研究中被进一步被证实^[59]^。

### 2.4 亚洲共识指南

2016年2月亚洲肺部疾病和胸外科多学科专家小组在美国胸科医师学会制定的肺结节评估指南的基础上结合亚洲患者的自身特点，制订了亚洲肺结节患者的评估指南^[60-62]^。亚洲肺结节的评估与APCC指南中所指出的重要注意事项大致相同，但该指南指出临床医生应该重视室内和室外空气严重污染导致的肺癌风险，还有女性非吸烟人群肺腺癌的高发。亚洲由于肺结核高发，不宜对于肺部结节用PET进行筛查，应采用非手术活检确诊和定期监测。此外，对于肉芽肿性疾病和其他感染性疾病在亚洲高发病率高也应引起注意^[37]^。总体应该考虑延长ACCP推荐的对肺结节的观察时间。总之，在亚洲不同国家对肺结节的评价指南存在差异，中国、韩国及日本都有本国的指南，而新加坡、印度和泰国这些没有全国性指南的国家则按照当地机构的规范对肺结节进行评价。在一项亚洲地区内的非官方调查中发现，有一些常见的临床实践指南与ACCP指南的建议相饽^[63]^。指南中强调亚洲的医生应将该共识常规用于临床实践，以提高对肺结节评估的一致性。

### 2.5 国内专家共识

#### 2.5.1 肺部结节诊治中国专家共识

2015年4月中华医学会呼吸病学分会肺癌学组和中国肺癌防治联盟专家组，根据我国实际情况更新了现有的文献综述和综合证据，并参考了美国胸科医师学会肺癌指南（第三版）中“肺癌指南发展的方法学”和中华医学会呼吸病学分会肺癌学组及中国肺癌防治联盟专家组制定的“原发性肺癌早期诊断中国专家共识”制定了本共识，并分别讨论了结节直径 ＞8 mm、直径≤8 mm和不同密度结节（实性结节与非实性结节）。之所以将结节直径界限值定为8 mm，是因为≤8 mm者在短时间内发展为恶性肿瘤的可能性相对较小，或肿瘤倍增时间较长，目前较难用影像学技术进行精确评估，也很难进行非手术活检。根据共识，应对肺结节患者进行恶性肿瘤的概率估计、影像学检查、评估各种替代管理的相关风险，并征求患者的意愿进行评估和管理^[64]^。

#### 2.5.2 孤立性肺结节的处理

2009年3月中国抗癌协会肺癌专业委员会召开了“第六届中国肺癌高峰共识会”，来自全国的50多位专家讨论了孤立性肺结节的临床问题，形成了孤立性肺结节处理之中国共识。共识一：孤立性肺结节指的是单一的、边界清楚的、影像不透明的、直径小于或等于3 cm、周围为含气肺组织所包绕的病变，没有肺不张、肺门增大或胸腔积液表现的肺部结节。共识二：根据SPN之直径，可将≥8 mm而≤3 cm的孤立性肺结节称为典型的SPN，将＜8 mm的肺部结节称为小结节。共识三：一旦发现肺部典型性孤立性结节。应采用经过验证的方法进行良恶性的判别，这些方法包括：影像学上的形态学分析、孤立性结节的倍增时间、PET-CT或动态增强CT扫描检查、恶性概率计算等。共识四：恶性几率＜3%为低几率。可采取影像学观察的策略，恶性几率在3%-68%应进一步检查，恶性几率 ＞68%应以胸腔镜辅助下或完全性的胸腔镜下的楔形切除为主，术中快速冰冻切片检查如为恶性，应行肺叶切除+系统性纵隔淋巴结清除术。共识五：肺小结节患者的处理以临床观察为主。在制定观察策略之前，可先将患者按有没有肺癌危险因素分为两组。肺癌危险因素指的是：有吸烟史；年龄超过60岁：有肺癌史或肺外其他癌病史^[65]^。

#### 2.5.3 肺亚实性结节影像处理专家共识

2015年4月中华放射学杂志刊登了亚实性肺结节影像学专家共识，专家组成员主要为影像科医生，他们参考了国内外大量文献、最新进展和多学科指南，综合了中国学者的研究结果和专家意见及我国实际情况和医疗环境，期望能成为临床工作的重要参考和依据。共识中特别指出有以下变化提示恶性肺小结节（small pulmonary nodules, GGN）：①GGN增大；②稳定并密度增高；③稳定或增大，并出现实性成分；④缩小但病灶内实性成分增大；⑤结节具备其他形态学的恶性征象。有以下变化提示良性GGN：①病灶形态短期内变化明显，无分叶或出现极深度分叶，边缘变光整或变模糊；②密度均匀，密度变淡；③随访中病灶缩小（密度没有增高）或消失；④随访中病灶迅速变大（倍增时间＜15 d）；⑤病灶长期稳定，但实性结节2年无变化提示良性并不适用于GGN，因处于原位腺癌（adenocarcinoma *in situ*, AIS）和肺微浸润腺癌（minimally invasive adenocarcinoma, MIA）阶段的GGN可以长期稳定，所以这里的长期需要更长的时间，但究竟多长时间稳定提示良性，还需要进一步更加深人的研究^[66]^。

## 3 小结与展望

综上所述，肺结节作为临床工作中的常见问题，历经数十年研究及数版临床处理指南修订，虽然已经日趋完善，但仍有许多问题没有达成共识。这些指南间之所以存在差异，与指南制定者的专业背景、所属地域、医院性质等密不可分，不同国家，甚至同一国家的不同地区肺癌的发生率也不相同，临床医生应该根据自己的实际情况选择适合的指南，在充分告知潜在风险和收益的基础上，为肺结节患者提供有效、经济的处理路径。此外，肺结节的诊治过程中除了应用LDCT筛查，还应建立科学和规范的评估模型及随访策略，提高良恶性鉴别的准确率，既要使肺癌患者及时得到治疗，又要尽量减少对良性结节患者的过度治疗，国内应开展前瞻性临床研究，为制定中国的肺结节诊疗指南提供循证医学的证据。

## References

[1] Ettinger DS (2012). Ten years of progress in non-small cell lung cancer. J Natl Compr Canc Netw.

[2] Siegel R, Naishadham D, Jemal A (2013). Cancer statistics, 2013. CA Cancer J Clin.

[3] DeSantis C, Naishadham D, Jemal A (2013). Cancer statistics for African Americans, 2013. CA Cancer J Clin.

[4] Brett GZ (1968). The value of lung cancer detection by six-monthly chest radiographs. Thorax.

[5] Frost JK, Ball WC Jr., Levin ML (1984). Early lung cancer detection: results of the initial (prevalence) radiologic and cytologic screening in the Johns Hopkins study. Am Rev Respir Dis.

[6] Flehinger BJ, Melamed MR, Zaman MB (1984). Early lung cancer detection: results of the initial (prevalence) radiologic and cytologic screening in the Memorial Sloan-Kettering study. Am Rev Respir Dis.

[7] Fontana RS, Sanderson DR, Taylor WF (1984). Early lung cancer detection: results of the initial (prevalence) radiologic and cytologic screening in the Mayo Clinic study. Am Rev Respir Dis.

[8] Kubik A, Parkin DM, Khlat M (1990). Lack of benefit from semi-annual screening for cancer of the lung: follow-up report of a randomized controlled trial on a population of high-risk males in Czechoslovakia. Int J Cancer.

[9] Oken MM, Hocking WG, Kvale PA (2011). Screening by chest radiograph and lung cancer mortality: the Prostate, Lung, Colorectal, and Ovarian (PLCO) randomized trial. JAMA.

[10] Gould MK, Donington J, Lynch WR (2013). Evaluation of individuals with pulmonary nodules: when is it lung cancer? Diagnosis and management of lung cancer, 3rd ed: American College of Chest Physicians evidence-based clinical practice guidelines. Chest.

[11] Swensen SJ, Silverstein MD, Ilstrup DM (1997). The probability of malignancy in solitary pulmonary nodules. Application to small radiologically indeterminate nodules. Arch Intern Med.

[12] Maeshima AM, Tochigi N, Yoshida A (2010). Clinicopathologic analysis of multiple (five or more) atypical adenomatous hyperplasias (AAHs) of the lung: evidence for the AAH-adenocarcinoma sequence. J Thorac Oncol.

[13] Lee HY, Choi YL, Lee KS (2014). Pure ground-glass opacity neoplastic lung nodules: histopathology, imaging, and management. AJR Am J Roentgenol.

[14] Naidich DP, Bankier AA, MacMahon H (2013). Recommendations for the management of subsolid pulmonary nodules detected at CT: a statement from the Fleischner Society. Radiology.

[15] MacMahon H, Austin JH, Gamsu G (2005). Guidelines for management of small pulmonary nodules detected on CT scans: a statement from the Fleischner Society. Radiology.

[16] Wood DE (2015). National Comprehensive Cancer Network (NCCN) Clinical Practice Guidelines for Lung Cancer Screening. Thorac Surg Clin.

[17] Research T, Aberle DR, Berg CD (2011). The National Lung Screening Trial: overview and study design. Radiology.

[18] Research T, Aberle DR, Adams AM (2011). Reduced lung-cancer mortality with low-dose computed tomographic screening. N Engl J Med.

[19] Detterbeck FC, Mazzone PJ, Naidich DP (2013). Screening for lung cancer: Diagnosis and management of lung cancer, 3^rd^ ed: American College of Chest Physicians evidence-based clinical practice guidelines. Chest.

[20] Vansteenkiste J, Crino L, Dooms C (2014). 2^nd^ ESMO Consensus Conference on Lung Cancer: early-stage non-small-cell lung cancer consensus on diagnosis, treatment and follow-up. Ann Oncol.

[21] Smith RA, Brooks D, Cokkinides V (2013). Cancer screening in the United States, 2013: a review of current American Cancer Society guidelines, current issues in cancer screening, and new guidance on cervical cancer screening and lung cancer screening. CA Cancer J Clin.

[22] Moyer VA, Force USPST (2014). Screening for lung cancer: U.S. Preventive Services Task Force recommendation statement. Ann Intern Med.

[23] Grutters JP, Kessels AG, Pijls-Johannesma M (2010). Comparison of the effectiveness of radiotherapy with photons, protons and carbon-ions for non-small cell lung cancer: a meta-analysis. Radiother Oncol.

[24] Palma D, Visser O, Lagerwaard FJ (2010). Impact of introducing stereotactic lung radiotherapy for elderly patients with stage Ⅰ non-small-cell lung cancer: a population-based time-trend analysis. J Clin Oncol.

[25] Shirvani SM, Jiang J, Chang JY (2012). Comparative effectiveness of 5 treatment strategies for early-stage non-small cell lung cancer in the elderly. Int J Radiat Oncol Biol Phys.

[26] Grills IS, Mangona VS, Welsh R (2010). Outcomes after stereotactic lung radiotherapy or wedge resection for stage Ⅰ non-small-cell lung cancer. J Clin Oncol.

[27] Crabtree TD, Denlinger CE, Meyers BF (2010). Stereotactic body radiation therapy versus surgical resection for stage Ⅰ non-small cell lung cancer. J Thorac Cardiovasc Surg.

[28] Dillman RO, Herndon J, Seagren SL (1996). Improved survival in stage Ⅲ non-small-cell lung cancer: seven-year follow-up of cancer and leukemia group B (CALGB) 8433 trial. J Natl Cancer Inst.

[29] Baumann M, Herrmann T, Koch R (2011). Final results of the randomized phase Ⅲ CHARTWEL-trial (ARO 97-1) comparing hyperfractionated-accelerated versus conventionally fractionated radiotherapy in non-small cell lung cancer (NSCLC). Radiother Oncol.

[30] Mauguen A, Le Pechoux C, Saunders MI (2012). Hyperfractionated or accelerated radiotherapy in lung cancer: an individual patient data meta-analysis. J Clin Oncol.

[31] Thomas M, Rube C, Hoffknecht P (2008). Effect of preoperative chemoradiation in addition to preoperative chemotherapy: a randomised trial in stage Ⅲ non-small-cell lung cancer. Lancet Oncol.

[32] Higgins K, Chino JP, Marks LB (2009). Preoperative chemotherapy versus preoperative chemoradiotherapy for stage Ⅲ (N2) non-small-cell lung cancer. Int J Radiat Oncol Biol Phys.

[33] Curran WJ Jr., Paulus R, Langer CJ (2011). Sequential *vs*. concurrent chemoradiation for stage Ⅲ non-small cell lung cancer: randomized phase Ⅲ trial RTOG 9410. J Natl Cancer Inst.

[34] Sause W, Kolesar P, Taylor SI (2000). Final results of phase Ⅲ trial in regionally advanced unresectable non-small cell lung cancer: Radiation Therapy Oncology Group, Eastern Cooperative Oncology Group, and Southwest Oncology Group. Chest.

[35] Eisenberg RL, Bankier AA, Boiselle PM (2010). Compliance with Fleischner Society guidelines for management of small lung nodules: a survey of 834 radiologists. Radiology.

[36] Travis WD, Brambilla E, Noguchi M (2011). International association for the study of lung cancer/american thoracic society/european respiratory society international multidisciplinary classification of lung adenocarcinoma. J Thorac Oncol.

[37] Godoy MC, Naidich DP (2009). Subsolid pulmonary nodules and the spectrum of peripheral adenocarcinomas of the lung: recommended interim guidelines for assessment and management. Radiology.

[38] Kim HK, Choi YS, Kim J (2010). Management of multiple pure ground-glass opacity lesions in patients with bronchioloalveolar carcinoma. J Thorac Oncol.

[39] Kim HK, Choi YS, Kim K (2009). Management of ground-glass opacity lesions detected in patients with otherwise operable non-small cell lung cancer. J Thorac Oncol.

[40] Jeudy J, White CS, Munden RF (2008). Management of small (3-5-mm) pulmonary nodules at chest CT: global survey of thoracic radiologists. Radiology.

[41] van Klaveren RJ, Oudkerk M, Prokop M (2009). Management of lung nodules detected by volume CT scanning. N Engl J Med.

[42] Goo JM, Park CM, Lee HJ (2011). Ground-glass nodules on chest CT as imaging biomarkers in the management of lung adenocarcinoma. AJR Am J Roentgenol.

[43] Bach PB, Kelley MJ, Tate RC (2003). Screening for lung cancer: a review of the current literature. Chest.

[44] Bach PB, Niewoehner DE, Black WC (2003). Screening for lung cancer: the guidelines. Chest.

[45] Pinsky PF (2004). An early-and late-stage convolution model for disease natural history. Biometrics.

[46] Hunt I, Siva M, Southon R (2006). Does lung cancer screening with chest X-ray improve disease-free survival?. Interact Cardiovasc Thorac Surg.

[47] Manser RL, Irving LB, Byrnes G (2003). Screening for lung cancer: a systematic review and meta-analysis of controlled trials. Thorax.

[48] Manser RL, Irving LB, Stone C (2004). Screening for lung cancer. Cochrane Database Syst Rev.

[49] Bach PB, Mirkin JN, Oliver TK (2012). Benefits and harms of CT screening for lung cancer: a systematic review. JAMA.

[50] Fontana RS, Sanderson DR, Woolner LB (1991). Screening for lung cancer. A critique of the Mayo Lung Project. Cancer.

[51] Blanchon T, Brechot JM, Grenier PA (2007). Baseline results of the Depiscan study: a French randomized pilot trial of lung cancer screening comparing low dose CT scan (LDCT) and chest X-ray (CXR). Lung Cancer.

[52] Garg K, Keith RL, Byers T (2002). Randomized controlled trial with low-dose spiral CT for lung cancer screening: feasibility study and preliminary results. Radiology.

[53] Gohagan JK, Marcus PM, Fagerstrom RM (2005). Final results of the Lung Screening Study, a randomized feasibility study of spiral CT versus chest X-ray screening for lung cancer. Lung Cancer.

[54] Lopes Pegna A, Picozzi G, Mascalchi M (2009). Design, recruitment and baseline results of the ITALUNG trial for lung cancer screening with low-dose CT. Lung Cancer.

[55] Infante M, Cavuto S, Lutman FR (2009). A randomized study of lung cancer screening with spiral computed tomography: three-year results from the DANTE trial. Am J Respir Crit Care Med.

[56] Saghir Z, Dirksen A, Ashraf H (2012). CT screening for lung cancer brings forward early disease. The randomised Danish Lung Cancer Screening Trial: status after five annual screening rounds with low-dose CT. Thorax.

[57] Bastarrika G, Garcia-Velloso MJ, Lozano MD (2005). Early lung cancer detection using spiral computed tomography and positron emission tomography. Am J Respir Crit Care Med.

[58] Henschke CI, Naidich DP, Yankelevitz DF (2001). Early lung cancer action project: initial findings on repeat screenings. Cancer.

[59] Menezes RJ, Roberts HC, Paul NS (2010). Lung cancer screening using low-dose computed tomography in at-risk individuals: the Toronto experience. Lung Cancer.

[60] Kim YK, Lee SH, Seo JH (2010). A comprehensive model of factors affecting adoption of clinical practice guidelines in Korea. J Korean Med Sci.

[61] She J, Yang P, Hong Q (2013). Lung cancer in China: challenges and interventions. Chest.

[62] Detterbeck FC, Lewis SZ, Diekemper R (2013). Executive Summary: Diagnosis and management of lung cancer, 3^rd^ ed: American College of Chest Physicians evidence-based clinical practice guidelines. Chest.

[63] Detterbeck FC, Homer RJ (2011). Approach to the ground-glass nodule. Clin Chest Med.

[64] Chinese medicine association respiratory disease branch lung cancer group, Chinese Lung Cancer Prevention Alliance expert group (2015). Disease Research Institute. China expert consensus of diagnosis and treatment of pulmonary nodule. Zhonghua Jie He Yu He Xi Za Zhi.

[65] Wu YL, Jiang GL, Liao ML (2009). Chinese consensus on treatment of solitary pulmonary nodule. Xun Zheng Yi Xue.

[66] Cardiothoracic society of Radiology branch of Chinese Medical Association (2015). Part solid lung nodules image processing expert consensus. Zhonghua Fang She Xue Za Zhi.

